# Stem Cell Therapy in the Management of Fracture Non-Union – Evaluating Cellular Mechanisms and Clinical Progress

**DOI:** 10.7759/cureus.13869

**Published:** 2021-03-13

**Authors:** Harman Khatkar, Abbas See

**Affiliations:** 1 Trauma and Orthopaedics, Royal Berkshire Hospital, Reading, GBR; 2 Nuffield Department of Orthopaedics, Rheumatology and Musculoskeletal Sciences (NDORMS), University of Oxford, Oxford, GBR; 3 Trauma and Orthopaedics, Kettering General Hospital, Kettering, GBR

**Keywords:** orthopaedics, orthopaedics surgery, stem cells, adipose derived stem cells, bone density

## Abstract

Bone, as a physiological and anatomical construct, displays remarkable intrinsic healing capacity. The overwhelming majority of fractures will heal satisfactorily, if aligned anatomically, compressed and immobilised appropriately. Of the 10% of fractures that do not heal, even under ideal mechanical and biological conditions, further consideration must be given to augment bone healing. Management strategies for non-union pose a significant clinical challenge to the practicing orthopaedic surgeon. Stem cell therapy is beginning to demonstrate significant potential for augmented bone repair in the context of non-union. This review attempts to contextualise the function of stem cells within this clinical setting, reviewing the relevant cellular mechanisms and clinical applications. From evaluating the literature base, there is a lack of high-quality evidence examining the role of mesenchymal stem cells (MSCs) within this research focus. Appropriately designed randomised controlled trials are required to evaluate this research area further, with a view to guiding future treatment options for the practicing orthopaedic surgeon.

## Introduction and background

Bone, as a physiological and anatomical construct, displays remarkable intrinsic healing capacity [1]. The overwhelming majority of fractures will heal satisfactorily, if aligned anatomically, compressed and immobilised appropriately [2, 3]. Of the 10% of fractures that do not heal, even under ideal mechanical and biological conditions, further consideration must be given to augment bone healing. Management strategies for non-union pose a significant clinical challenge to the practicing orthopaedic surgeon. A widely-accepted concept for augmenting bone healing in the non-union clinical setting involves employing the ‘diamond concept’; attempting to ensure a combination of an optimal mechanical environment, growth factors, osteoconductive scaffolds and stem cells [3].

Stem cell therapy is beginning to demonstrate significant potential for translational clinical application, across a variety of surgical and medical specialties. Both in vitro and in vivo experimental models have demonstrated promising results; positive therapeutic effects relating to augmented bone repair in the context of disease have been produced [1, 3].

This review aims to explain what a stem cell is, relating this to their physiological function and highlighting the variety of stem cells present, whilst discussing methods of stem cell isolation for use in bone repair models. This review will also aim to evaluate the fundamentals of bone repair at the cellular level, focusing on the context of fracture non-union. An insight into future stem cell application in bone repair and disease will also be included, with limitations of the current evidence base also explored. We also hope this paper serves as a useful primer and aide-memoire for the practicing orthopaedic surgeon on the evolving concepts of bone healing and stem cell therapy in the context of non-union.

## Review

Methodology

A focused literature search was performed using the Pubmed/MEDLINE index. Inclusion criteria included the use of human studies, papers written in the English language and full papers. Published abstracts, wholly non-human studies and non-English language papers were excluded. All relevant literature was screened for inclusion, with relevant papers proceeding to further full screen evaluation.

Stem cell biology

To evaluate the advances in stem cell therapy relating to bone repair and disease, the fundamentals of stem cells must be explained to provide clinical context. 'Stemness' is a term used to describe a cell with stem-cell qualities compared to a non-stem cell [4]. A succinct definition of a stem cell is that of a self-renewing cell that can differentiate in a symmetric and asymmetric fashion. This is to say that a stem cell can divide by clonal expansion leading to the production of more of the same stem cells and it can also differentiate into different cell progeny [5].

Concerning stem cell potency, a 'true' stem cell is multipotent, differentiating into more than one cell type. In contrast, a progenitor cell derives from a multipotent stem cell but is unipotent, dividing into a single progeny [6]. Dependent upon the type of stem cell, it can demonstrate pluri-, toti-, multi- or unipotent developmental potential [7]. Table 1 demonstrates definitions of these specific terms in relation to cell potency [3, 8, 9].

**Table 1:** Definition of specific terms relating to cell potency

	Totipotent	Pluripotent	Multipotent	Unipotent
Relative Potency	High	Medium	Low	Low
Examples	- Fertilised ovum	- Embryonic Stem Cells - Induced Pluripotent Stem Cells	- Haematopoietic Stem cells	- All cell types
Developmental potential	Any cell type	Can differentiate into cell types from 3 primordial germ layers	Differentiate into limited range of cell types	Can only give rise to one single cell type

Self-renewal describes the mode of proliferation specific to stem cells. For a cell to be self-renewing, it must produce a daughter cell with the same replicative potential of the mother cell. An example of this type of cell lineage are neural stem cells, where both daughter and mother cells are multipotent [10].

Types of stem cell

Stem cells can be broadly divided into two categories: those of embryonic origin and those of non-embryonic origin. Human embryonic stem cells (ESCs) are considered the ‘gold standard’ for developing understanding of stem cell pluripotency [11]. ESCs are obtained from the blastocyst stage of the embryo; cells obtained are pluripotent. Undifferentiated stem cells have also been sourced [12]. ESCs can differentiate into any cell derived from the three primordial germ layers, thus can be considered pluripotent [2]. Human embryonic stem cell research remains an area of intense ethical and political scrutiny, given the method of harvest ultimately requires embryonic destruction [3]. A variety of other stem cells from a non-embryonic origin exist, such as amniotic epithelial cells and fetal stem cells, however thorough explanation of these is beyond the scope of this review [13].

The focus of this review concerns adult stem cells (ASCs). An example of these are haematopoietic stem cells (HSCs), which have the capacity to derive all types of mature blood cell [14]. There are a range of cell types in this category, but this review will focus on stem cells which can differentiate into tissues of a mesodermal origin and assist in bone repair and disease. Cells of a mesodermal origin include chondrocytes, muscle cells, adipocytes and skeletal tissue cells [15].

One stem cell population that derives the aforementioned cell types are mesenchymal stem cells (MSCs), cells found primarily in adult bone marrow [16]. MSCs are low in number compared to the rest of the cell population in the bone marrow and they originate primarily from the stromal cell system; a system of non-haematopoietic connective tissues [17]. The mesenchyme is mesodermal-derived tissue that can develop into a variety of connective tissue types. The isolation of MSCs requires a bone marrow aspiration from the superior iliac crest of the donor. This is then centrifuged to separate the mononuclear layer of cells and then this cell layer is cultured to allow the MSCs present to adhere to a plastic surface, enabling extraction [18-20]. The integral clinically relevant property of MSCs is their ability to differentiate into various types of connective tissue, and their ability to sustain self-renewal. MSC cultures must be exposed to a different combination of substances to produce different cell populations. Table 2 highlights these combinations [18].

**Table 2:** Conditions needed for MSC differentiation into different cell types TGF: Transforming growth factor; PPAR: Peroxisome proliferator-activated receptor; EGF: Epidermal growth factor; MSC: Mesenchymal stem cell.

Cell type	Conditions
Chondrocyte	Requires TGF-beta, a nutrient medium which does not contain serum and a three-dimensional culture zone
Osteoblast	Requires the presence of fetal bovine serum, ascorbic acid-2-phosphate, beta-glycerol-phosphate and dexamethasone
Adipocyte	Requires PPAR-gamma and fatty acid synthetase
Neuron	Has been shown to occur with the presence of isobutylmethylxanthine and dibutyrul cyclic AMP. Also shown to occur with the presence of brain-derived neurotrophic factor and EGF.

Concerning clinical musculoskeletal application, the scope of use is far-reaching [3, 13, 21]. Pre-clinical animal models have demonstrated that an intramuscular injection of MSCs into murine muscle can help treat Duchenne's muscular dystrophy [22]. Translational clinical benefit has been demonstrated by autologous transplantation of MSCs of a specified myogenic lineage in patients with Duchenne’s muscular dystrophy, successfully increasing the amount of dystrophin-competent cells [23]. The therapeutic effects of MSCs in graft-versus-host disease (GVHD) have been extensively studied and demonstrate the role of MSCs in immunomodulation. Studies have illustrated that MSC administration diminishes the immune response associated with GVHD by modifying specific unwanted actions of immune cells, thus decreasing chances of organ rejection [24]. Multiple further clinical uses of MSCs have been demonstrated, however their application in the fracture non-union setting appears less fully understood [3].

Mesenchymal stem cells are introduced into the clinical site either by direct injection into the clinical site, or along bioengineered scaffolds which seek to promote proliferation, MSC survival and are considered more osteoconductive surfaces in comparison to the host surface [25].

Principles of normal bone repair and maintenance: fracture healing

Consolidation of a fracture site occurs through a combination or primary (direct) bone healing, secondary (indirect) bone healing, or a combination of both mechanisms. This can be augmented by micro motion at the fracture site, however an overload of motion can lead to non-union of the fracture [26]. A widely-accepted biomechanical concept governing fracture stability pertains to the fracture site accepting 2-10% strain, with strain applied outside of these margins promoting non-union [27].

Direct bone healing (primary healing) occurs when fracture fragments are compressed and are directly opposed. Compression is produced by surgical implantation, for example dynamic compression plating, or by immobilisation (for example splinting or cast immobilisation). Implants directly compress the fracture fragment, which on mechanical loading stimulates bone formation allowing the joint to bear load as time progresses. Initially the implant bears mechanical load, but as healing progresses and more bone is laid down, load bearing is transferred gradually to newly formed bone. Osteoclasts and osteoblasts produce bone by forming constructs known as ‘cutting cones’, whereby osteoclasts resorb bone and osteoblasts deposit bone following this. Only bone present at the implant-bone interface is restored with this method. Repair needs to occur outside of the interface to ensure mechanical integrity of the construct, thus a process known as gap healing occurs, whereby pockets of fractured bone not at the interface are repaired by periosteal osteoblastic activity. Approximately 12 weeks after implantation the newly formed bone acquires the tensile strength of normal bone [28]. If the fracture ends are not in continuity or under sufficient compression, an external callus normally forms at the fracture site, in the presence of a fracture haematoma which acts as a nutrient medium, osteoconductive scaffold and fibrin mesh for subsequent bone deposition. This differentiates through various connective tissue forms until bone is formed by osteoblasts. Fragile woven bone created by this process is remodelled into stronger lamellar bone. This can be considered the classical process whereby secondary (indirect) bone healing occurs [2, 26, 29].

Within the quoted literature, fracture healing is dependent principally upon five conditions at the fracture site. These include there being a sufficient blood supply, a fracture-haematoma, a population of cells diverse enough to enhance healing and provide nutrition to the site, a constant mechanical environment, and an osteoinductive scaffold for repair to occur on [1-3, 26].

Fracture non-union: definition and aetiology

For the purposes of the practicing orthopaedic surgeon, a non-union is a fracture that will not heal without further intervention [30]. The causes of non-union can be broadly categorised into three separate categories. As per the work of Stewart, these can be summarised as host factors, biological factors and mechanical factors [31]. Host factors well validated within the literature include smoking, diabetes, immunosuppression, compliance with post-operative advice and steroid use. Biological factors include presence of active infection, adequacy of soft tissue coverage, bone defect and vascular supply to the fracture site. Mechanical factors include fracture configuration and adequacy/suitability of surgical fixation [21, 31]. Clinically, patients with established non-union report persistent pain around the fracture site, with the possible awareness of fracture fragment motion, and reduced functional capacity of the affected limb [31, 32].

Distinguishing between different types of non-union is important for clinical practice and guides surgical management. The classification of morphological type of non-union allows the clinician to target treatment appropriately. Non-union can be classically divided into hypertrophic union, whereby the fracture ends are viable and capable of mounting biologic reaction, and atrophic, whereby fracture ends are inert and incapable of mounting a biologic reaction [33]. The accepted method of classification divides non-unions by cause and fracture configuration into further sub divisions [34]. See Table 3 for further sub divisions [29, 33, 35].

**Table 3:** Types of non-union based on morphological classification of fracture site

Type of non-union	Hypertrophic vs Atrophic	Causes	Characteristics
Elephant foot	Hypertrophic	Joint mobilisation and weight bearing before the repair process has a chance to start	Hypertrophic and lots of callus present
Horse foot	Hypertrophic	After ineffective surgery involving poor fixation with implants	Slightly hypertrophic and very little callus present
Oligotrophic	Some debate exists – cannot be definitively considered atrophic/hypertrophic	Fracture is significantly displaced	Some vascularity exists with some callus present but no evidence of sufficient bridging callus
Torsion wedge	Atrophic	Occur after the treatment of tibial fractures involving implants	Presence of a small fracture fragment which has a poor blood supply
Comminuted	Atrophic	Occur due to failing and breaking of implants used for internal fixation	Presence of one or more necrotic fracture fragments
Defect	Atrophic	Occur due to fracture or through infection	Loss of a small part of the diaphysis. As time progresses, fracture fragments become atrophic
Atrophic	Combination of torsion wedge, comminuted and defect – atrophic	End-stage result of torsion wedge, comminuted and defect	Fragments of fracture are missing, scar tissue presence, lack of osteogenesis, limb inactivity has led to atrophy.

Management of non-union is dependent on complex decision making relating to surgical, implant and patient factors. Broadly speaking, hypertrophic non-union is treated by ensuring adequacy of mechanical stability at the fracture site. As the fracture site is considered biologically active, callus formation will be considered sufficient without external modification [31, 36]. Atrophic non-union management has been principally focused on restoring a normal biological environment for bone production to occur, with a focus on autologous bone grafts (ABG) to bridge segmental bone defects. The use of MSCs in the context of atrophic non-union presents an alternative viable therapeutic option for such defects [3, 31].

Preparation and conditioning of MSCs

The successful differentiation of MSCs to osteoblasts is the basis of potential treatment options in non-union; autologous stem cells could serve to provide a native osteoblast population for the recipient, stimulating bone growth at the fracture site.

The preparation and conditioning of MSCs is fundamental in facilitating differentiation into osteoblasts. Historically, the vital factors governing successful differentiation were considered scaffold composition, the surface scaffold topography and the interaction between the stem cell and the scaffold.

Two approaches to conditioning stem cells specifically down an osteoblastic lineage have been demonstrated in the literature: the ‘top-down’ and ‘bottoms-up’ approaches [37]. The first approach is considered a ‘top-down’ approach, whereby bioengineering focus is placed on optimising the extracellular environment to augment MSC differentiation down. Success has been demonstrated by including the combination of MSCs with hydroxyapatite (HA) granules and bone morphogenetic protein-2 (BMP-2), to promote osseointegration by utilising the biocompatibility and porosity of the added materials [32, 38]. Dilogo et al. appear to have demonstrated effective clinical results in promoting fracture healing and scaffold integration by utilising MSC and a ‘top-down’ approach in treating segmental bone defects in the non-union setting, utilising MSCs, HA granules and BMP-2 to facilitate both an osteogenic and osteoinductive environment [38]. The ‘top-down’ approach can be considered the more well-validated tissue engineering technique in stem cell differentiation setting [37].

As discussed by Kim and Mikos, the use of the ‘top-down’ approach invariably incorporates the use of external factors such as BMP-2 at supra-physiological levels, leading to possible complications reported as over-activation of osteoclasts, heterotopic ossification and autoimmune dysregulation [37]. To bypass these effects, the ‘bottoms-up’ approach can be considered. The ‘bottoms-up’ approach focuses on genetic modification to MSCs, leading to regulation of gene expression and thus theoretically controlling differentiation at the intrinsic genetic level. Novel gene delivery vehicles are currently being evaluated for their proposed efficacy in providing stable genetic modification for MSCs. The complex bioengineering concepts underpinning this are beyond the scope of this review.

A further growth-area focuses on the isolation of specific MSC sub-populations [39]. MSCs can be considered an inherently heterogeneous cell population. Isolating surface markers and morphological characteristics, for example cell size, of MSCs that have successfully undergone osteoblastic differentiation could allow for a more reliable and robust differentiation process [37, 39].

Clinical applications

The first reported clinical use of stem cell engineering in fracture repair was carried out by Vacanti et al. in 2001 [40]. A patient with an avulsed digital phalanx of the hand received stem cell therapy in addition to traditional reconstructive surgical techniques. The traditional reconstructive techniques of splinting and skin grafting occurred. Additionally, a pocket of periosteal cells was introduced into the surgical site on a bioengineered scaffold. The patient reported a successful functional outcome at three months. The use of periosteal cells to augment bone healing at the surgical site has led to further focused clinical work in this field [29, 40]. The work of Quarto et al. can be considered the seminal work in stem cell engineering and non-union [41]. Bone marrow was extracted from patients suffering from significant segmental bone defects. This was then cultured ex vivo and the MSCs present in the bone marrow differentiated into osteoprogenitor cells, which were then placed on a bio-engineered scaffold and transferred back to the patients. Abundant radiological callus formation was demonstrated, alongside functional recovery. Quarto et al. did not explicitly state the context of non-union but given the post traumatic setting and subsequent bone loss, the non-union is likely to be considered atrophic in nature and thus would be more appropriate for biologic augmentation [41].

Clinical studies have highlighted that patients injected with bone marrow of which the MSC concentration was less than 1000 MSCs/ml did not experience augmented bone growth, whereas patients injected with bone marrow aspirate of which the MSC concentration was around 1500 MSCs/ml experienced rapid healing [3, 41]. There does not appear to be an agreed consensus on the optimal concentration for inducing bone growth, with further clinical work required to ascertain the appropriate concentration for inducing augmented bone healing. Reflecting upon the five conditions for fracture healing highlighted earlier, each one needs to be fulfilled for bone repair to occur. An issue of concern relates to the fact that MSC numbers obtained from a bone marrow aspiration are highly variable from patient to patient, thus providing an effective, reproducible treatment is proving difficult. This variability can be overcome by isolating a small number of MSCs and allowing them to proliferate ex vivo, but this method of treatment is logistically difficult, technically demanding, and costly. Bioengineering solutions have been theorised to negate a lack of MSCs, including genetically modifying MSCs to over express BMP, leading to an enhanced osteogenic effect of each MSC. Another possible future approach involves implanting a scaffold with MSCs into a highly vascularised tissue like skeletal muscle, allowing the scaffold to grow in vivo and obtain sufficient vascularisation and growth factors. This could then be transferred to the site of non-union [42]. These future challenges present the next exciting research chapter in the bone repair field [21].

Multiple innovative surgical strategies have been developed for bridging bone defects, such as vascularised bone grafting, employing the Ilizarov technique and the Masquelet technique [43]. One such technique that has had limited success using stem cell therapy is the Masquelet technique. In summary; the Masquelet technique involves the use of a temporary cement spacer to bridge the bone defect, followed by staged grafting with non-vascularised cancellous bone graft [44]. This technique takes advantage of the ‘induced membrane’, whereby a membrane forms around the temporary spacer, allowing theoretically for low bone graft resorption, anatomical bridging of defect and an absence of invasion by surrounding soft tissues [43]. At the time of bone grafting, native stem cell populations have been introduced into the fracture site, demonstrating successful bone healing in an isolated case by Dilogo et al. [45]. The role of stem cells in the context of the Masquelet technique appears to be an area that requires further research development, realising the potential of the induced membrane effect to enhance the efficacy of MSCs, by theoretically providing a suitably osteogenic and inductive environment for bone formation. To date, no robust clinical data evaluating the role of stem cells in comparison to control subjects is available, with research into the Masquelet technique still relatively underdeveloped, in comparison to the well-established techniques of vascularised bone grafting and the Ilizarov technique.

Another potential therapeutic option involving a different population of human stem cell has also been explored clinically. The use of human multipotent adipose-derived stem cells (hMADSCs) could potentially counteract some of the problems associated with isolation of MSCs. Obtaining hMADSCs from adipose tissue is non-invasive, and adipose tissue is generally abundant in-patient populations. Adipose-derived stem cells are also considered to have a high degree of cellular plasticity; meaning they can differentiate into a variety of cell populations [46]. One study illustrated that Adipose derived stem cells (ASCs) injected into a murine model with non-union fractures provided a substantial bone growth. At the initial stages of fracture consolidation, the ASCs differentiated into cells of haematopoietic and osteoblastic lineage. A further novel effect was demonstrated, whereby ASCs induced a local paracrine effect on the fracture site that stimulated osteogenesis and angiogenesis [47]. Veriter et al. have demonstrated positive effects clinically using hMADSCs to augment bone repair in critical bone defects, either due to oncological resection or congenital defect [46]. Grafts were developed with incorporated ASCs, alongside demineralised bone matrices, which were then incorporated into the site of defect. Four-year follow-up demonstrated osseointegration and biological integration of the grafts with evidence of union radiologically. These results, albeit promising, are not reflected in the underdeveloped research base relating to ASCs in clinical use. Concerns remain relating to immunomodulation and possible immunosuppressive capacity of ASCs; mechanisms relating to possible tumorigenicity have been proposed [48].

Percutaneous autologous bone-marrow grafting has now been used in several studies with success, illustrating that MSCs present in extracted bone marrow are playing a useful therapeutic role in aiding the repair of a fracture. A correlation has also been uncovered relating to the efficacy of the treatment and the number of osteoprogenitor cells present in the graft, merely underlining the importance of the initial stem cells in the treatment process [35]. Further promising clinical trials have been conducting using percutaneous bone marrow for long bone reconstruction, with injection performed under fluoroscopic guidance [49]. Emadedin et al. demonstrated a safe and effective approach by utilising percutaneous autologous bone marrow-derived MSC implantation in long bone atrophic union, but further randomised double-blind trials are required to fully assess effect-size and safety of this orthobiologic approach [49].

Strengths and limitations

Significant clinical limitations exist relating to the long-term efficacy of MSCs in the treatment of non-union. Whilst a short-term positive clinical efficacy has been demonstrated by recent work, long-term 5-10 year follow-up of patients has not been evaluated within the current literature base [3, 21, 31, 38]. Long-term follow-up analyses of current patient cohorts are needed to assess the anatomical and functional integrity of any bone repair, and whether any adverse effects of MSC use can be identified in the medium-to-long-term follow-up period.

Further, undertaking MSC research in this context appears to require translational academic expertise in musculoskeletal tissue engineering and orthopaedic surgery. Barriers to access are reflected in the paucity of robust translational literature, with most research produced in tertiary academic centres currently. Further to this end, there appears to be distinct heterogeneity relating to method of obtaining stem cells, isolation, and subsequent introduction into the defect. Such heterogeneity makes comparison between methods challenging.

Critically, there does not appear to be an internationally agreed definition for ‘non-union’, either defined through radiological or clinical parameters. Thus, production of robust meta-analyses and quantitative analysis of stem cell use within this setting would appear unfeasible, given seemingly equivalent studies would have inherent methodological heterogeneity. Heterogeneity in the assessment of fracture consolidation makes discerning whether MSCs are clinically beneficial difficult, with direct comparison between study outcomes poor due to this lack of uniformity in outcome measurement. Outcome measurement should focus on not just radiological outcome, but patient reported outcomes, combining holistic measurement of pain and functional status [3].

Further, prospective cohort analysis appears to be the prevailing study methodology, with no control subjects appearing within the scope of any such trial of MSC application in non-union. Without control subjects, examining the true effect of MSCs is challenging both from a clinical and methodological standpoint.

## Conclusions

This review references and appraises the key literature relating to stem cell usage in the non-union setting, contextualising the key research advances and areas of research focus. We have also summarised the salient clinical and basic science literature relating to bone repair at the cellular level. In summary, the role of MSCs in the treatment of non-union does not appear to be fully understood. Understanding surrounding isolation, culture, and implantation of stem cells in the context of non-union is based on principally heterogenous case studies, lacking randomisation and homogenous research techniques. There is a lack of high-quality evidence examining the role of MSCs within this research focus. Appropriately designed randomised controlled trials are required to evaluate this research area further, with a view to guiding future treatment options for the practicing orthopaedic surgeon.
